# Investigation of Antioxidative and Anticancer Potentials of *Streptomyces* sp. MUM256 Isolated from Malaysia Mangrove Soil

**DOI:** 10.3389/fmicb.2015.01316

**Published:** 2015-11-26

**Authors:** Loh Teng-Hern Tan, Hooi-Leng Ser, Wai-Fong Yin, Kok-Gan Chan, Learn-Han Lee, Bey-Hing Goh

**Affiliations:** ^1^Biomedical Research Laboratory, Jeffrey Cheah School of Medicine and Health Sciences, Monash University MalaysiaBandar Sunway, Malaysia; ^2^Division of Genetics and Molecular Biology, Institute of Biological Sciences, Faculty of Science, University of MalayaKuala Lumpur, Malaysia

**Keywords:** *Streptomyces sp*., antioxidant, anticancer, Malaysia, mangrove

## Abstract

A *Streptomyces* strain, MUM256 was isolated from Tanjung Lumpur mangrove soil in Malaysia. Characterization of the strain showed that it has properties consistent with those of the members of the genus *Streptomyces*. In order to explore the potential bioactivities, extract of the fermented broth culture of MUM256 was prepared with organic solvent extraction method. DPPH and SOD activity were utilized to examine the antioxidant capacity and the results have revealed the potency of MUM256 in superoxide anion scavenging activity in dose-dependent manner. The cytotoxicity of MUM256 extract was determined using cell viability assay against 8 different panels of human cancer cell lines. Among all the tested cancer cells, HCT116 was the most sensitive toward the extract treatment. At the highest concentration of tested extract, the result showed 2.3-, 2.0-, and 1.8-folds higher inhibitory effect against HCT116, HT29, and Caco-2 respectively when compared to normal cell line. This result has demonstrated that MUM256 extract was selectively cytotoxic toward colon cancer cell lines. In order to determine the constituents responsible for its bioactivities, the extract was then subjected to chemical analysis using GC-MS. The analysis resulted in the identification of chemical constituents including phenolic and pyrrolopyrazine compounds which may responsible for antioxidant and anticancer activities observed. Based on the findings of this study, the presence of bioactive constituents in MUM256 extract could be a potential source for the development of antioxidative and chemopreventive agents.

## Introduction

Cancer is a common cause of mortality in the world population. Recently, American Cancer Society has reported that cancer as the second leading cause of death is expected to surpass cardiovascular disease in a few year times (Siegel et al., 2015). Furthermore, the incidence of the development of resistance to chemotherapy has become a major health problem (Riganti et al., 2015). This issue is more serious in economically less developed countries due to the lack of accessibility to standard diagnostic facilities and high cost of treatment (Jemal et al., 2010). Thus, there is an urgent need to search for alternative anticancer agents which may overcome the failure of chemotherapy. Free radicals are known to be the major etiology of a number of diseases such as coronary heart disease, degenerative diseases and cancer (Devasagayam et al., 2004). Although oxidation is an important biological process for energy generation in living organisms, the excessive free radical production and low antioxidant defense lead to oxidative stress which is detrimental to cells and also strongly associated with cancer development involving oxidative DNA damage. Due to the destructive role of free oxygen radicals, there are several cellular mechanisms involve in the eradication of the free radicals including the enzymatic conversion of reactive oxygen species (ROS, H_2_O_2_, ${\text{O}}_{2}^{-}•$, and •OH^−^) into less reactive species, chelation by transition metal catalysts as well as detoxification of ROS by antioxidants (Valko et al., 2006). Many synthetic antioxidant such as butylated hydroxycanisole, butylated hydroxytoluene and propyl gallate have been developed in order to retard oxidation process and prevent the progression of diseases caused by ROS (Maxwell, 1995). However, these synthetic antioxidative compounds which exhibited strong radical scavenging activity have been reported to cause severe side effects (Baardseth, 1989; Tepe et al., 2005). Thus, alternative antioxidants from natural sources are more preferable and many recent studies have shown that besides plants as rich source of antioxidants (Wong et al., 2012; Tan et al., 2015), microorganisms can be used for the production of natural antioxidants. Recently, many studies reported that mangrove *Streptomyces* produced antioxidative agents (Rao and Rao, 2013; Ser et al., 2015a).

The intertidal coasts in the tropical and subtropical coastal regions consist of an exclusive woody plant area known as the mangrove area. The mangrove ecosystem is among the world's most prolific environments and produces commercial forest products, supports coastal fisheries and protects the coastlines. These ecosystems are favorable habitats of a variety of flora and fauna of marine, freshwater and terrestrial species (Jennerjahn and Ittekkot, 2002). Factors such as salinity and tidal gradient in the mangrove systems are considered as some of the driving forces for metabolic pathway adaptations that could direct to the production of valuable metabolites (Hong et al., 2009; Lee et al., 2014d). Therefore, in recent years, there has been increasing interest in exploitation of mangrove microorganism resources. Furthermore, many researchers have successfully discovered novel actinobacteria strains from mangrove environments across the earth, such as the isolation of *Streptomyces avicenniae* (Xiao et al., 2009), *Streptomyces xiamenensis* (Xu et al., 2009), *Streptomyces sanyensis* (Sui et al., 2011), *Streptomyces qinglanensis* (Hu et al., 2012), *Streptomyces pluripotens* (Lee et al., 2014c), *Streptomyces mangrovisoli* (Ser et al., 2015a), and *Streptomyces gilvigriseus* (Ser et al., 2015b).

The genus *Streptomyces* was proposed by Waksman and Henrici (1943) and this genus is comprised of ca. 600 species with validly published names (http://www.bacterio.cict.fr/) at the time of writing (August 2015). Many members of *Streptomyces* have made imperative contributions to human with their capabilities to produce various important natural products (Bérdy, 2005). To date, numerous bioactive compounds with profound impact on society have been reported from the genus *Streptomyces* whereby over 7000 bioactive compounds with diverse bioactivities including antimicrobial, antioxidant, anticancer and antifungals properties are identified from *Streptomyces*. Beyond the well-known antibiotics from *Streptomyces*, such as streptomycin (Schatz et al., 1944) and erythromycin (Weber et al., 1985), many other medically useful agents include anticancer drugs such as doxorubicin (Grimm et al., 1994) and bleomycin (Du et al., 2000), the antifungal nystatin (Brautaset et al., 2000) are derived from *Streptomyces* as well. The unique and highly dynamic mangrove ecosystem is believed to exert significant influence on bacterial speciation for metabolic and physiological adaptations, consequently leading to the production of unique secondary metabolites with interesting bioactivities (Duncan et al., 2014; Lee et al., 2014d). Several previous studies on secondary metabolites from mangrove *Streptomyces* have documented a number of unique bioactive compounds. For instance, seven azlomycin F analogs, macrocyclic lactones, with anticancer and antibacterial properties were discovered recently from *Streptomyces* sp. 211726 isolated from mangrove rhizosphere soil (Yuan et al., 2013). Furthermore, benzonaphthyridine alkaloid was isolated from a mangrove-derived *S. albogriseolus* (Li et al., 2010). Fu and colleagues also revealed two indolocarbazoles, streptocarbazoles A and B with antitumor properties from *Streptomyces* sp. isolated from mangrove soil in Sanya, China (Fu et al., 2012).

In this study, *Streptomyces* sp. MUM256, isolated from soil at the Tanjung Lumpur mangrove forest, Peninsular Malaysia, was studied in the search of antioxidant and anticancer biological activities. The chemical constituents present in the extract of MUM256 were further characterized. The outcomes derived from this research constitute important starting points for performing further in depth biological studies focusing on free-radical associated diseases such as cancer.

## Materials and methods

### Isolation and maintenance of isolate

Strain MUM256 was isolated from a soil sample collected at site MUM-KS1 (3° 21′ 45.8″ N 101° 18′ 4.5″ E), located in the mangrove forest of Kuala Selangor in the state of Selangor, Peninsular Malaysia, in Jan 2015. Topsoil samples of the upper 20-cm layer (after removing the top 2–3 cm) were collected and sampled into sterile plastic bags using an aseptic metal trowel, and stored at −20°C. Air-dried soil samples were ground with a mortar and pestle. Selective pretreatment of soil samples was performed using wet heat in sterilized water (15 min at 50°C) (Takahashi et al., 1996). Five grams of the pretreated air-dried soil was mixed with 45 ml sterilized water and mill ground, spread onto the isolation medium ISP 2 (Shirling and Gottlieb, 1966) supplemented with cycloheximide (25 μg ml^−1^) and nystatin (10 μg ml^−1^), and incubated at 28°C for 14 days. Pure cultures of strain MUM256 were isolated and maintained on slants of ISP 2 agar at 28°C and as glycerol suspensions (20%, v/v) at −20°C for long term preservation.

### Genomic and phylogenetic analyses

**Figure 1:**
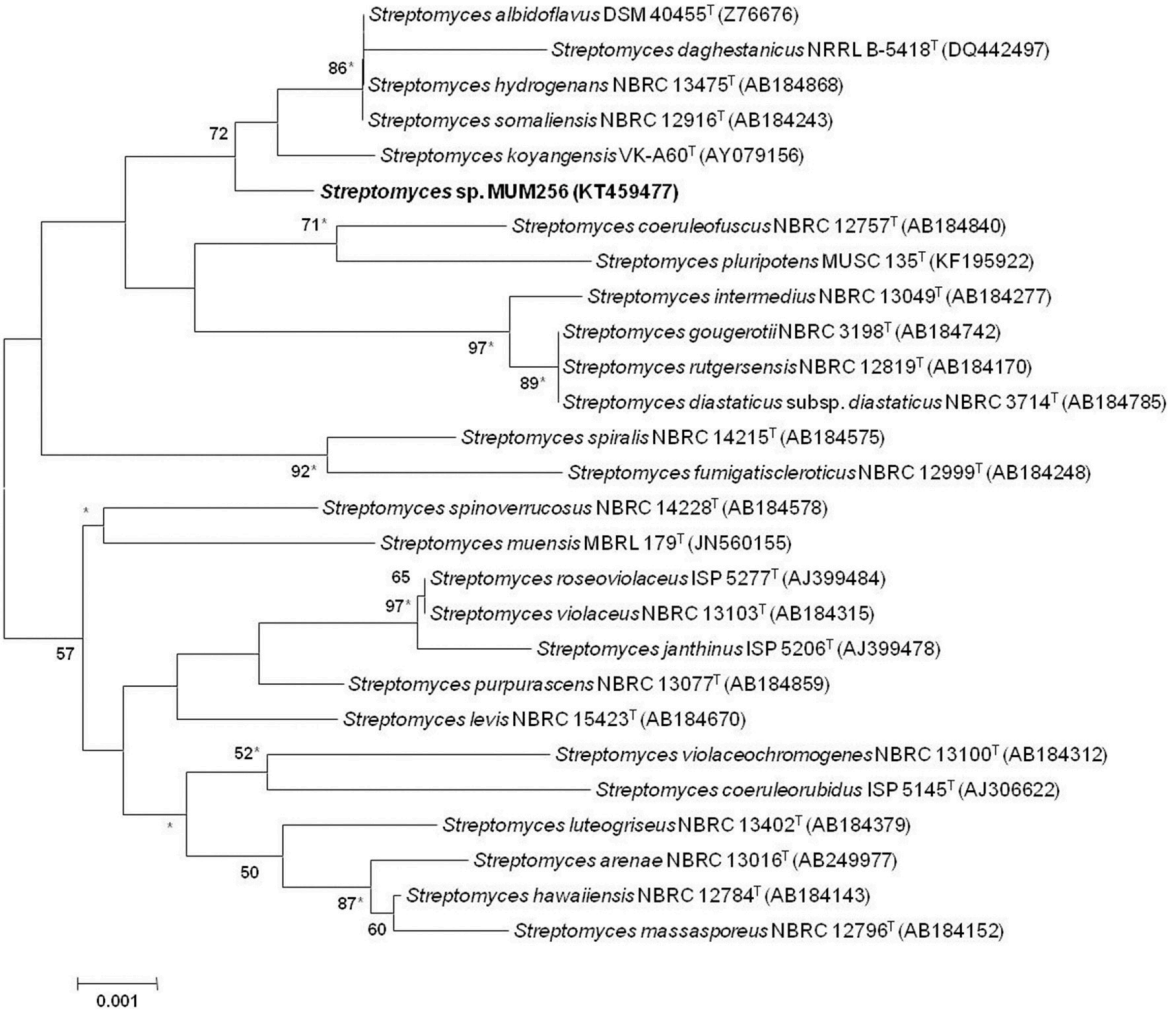
**Neighbor-joining phylogenetic tree based on 1487 nucleotides of 16S rRNA gene sequence showing the relationship between strain MUSC 149^***T***^ and representatives of related taxa**. Numbers at nodes indicate percentages of 1000 bootstrap re-samplings, only values above 50% are shown. Bar, 0.001 substitutions per site. Asterisks indicate that the corresponding nodes were also recovered using the maximum-likelihood tree-making algorithm.

The extraction of genomic DNA for PCR was performed as described by Hong et al. (2009). The amplification of 16S rRNA gene was performed according to Lee et al. (2014c). Briefly the PCR reactions were performed in a final volume of 50 μl according to protocol of SolGent™ 2X Taq PLUS PCR Smart mix using the Kyratex PCR Supercycler (Kyratec, Australia) with the following cycling conditions: (i) 95°C for 5 min, (ii) 35 cycles of 94°C for 50 s, 55°C for 1 min and 72°C for 1 min 30 s; and (iii) 72°C for 8 min. The 16S rRNA gene sequence of strain MUM256 was aligned with representative sequences of related type strains of the genus *Streptomyces* retrieved from the GenBank/EMBL/DDBJ databases using CLUSTAL-X software (Thompson et al., 1997). Phylogenetic trees were constructed with the neighbor-joining (Saitou and Nei, 1987; Figure 1) and maximum-likelihood (Felsenstein, 1981) and (Figure S1) algorithms using MEGA version 6.0 (Tamura et al., 2013). Evolutionary distances for the neighbor-joining algorithm were computed using Kimura's two-parameter model (Kimura, 1980). The EzTaxon-e server (http://eztaxon-e.ezbiocloud.net/; Kim et al., 2012) was used for calculations of sequence similarity. The stability of the resultant trees topologies were evaluated by using the bootstrap based on 1000 resampling method of Felsenstein (1985).

### Phenotypic characteristics

The cultural characteristics of strain MUM256 were determined following growth on ISP 2, ISP 3, ISP 4, ISP 5, ISP 6, ISP 7 (Shirling and Gottlieb, 1966), actinomycetes isolation agar (AIA) (Atlas, 2010), starch casein agar (SCA) (Küster and Williams, 1964), and nutrient agar (Mac Faddin, 1976) for 14 days at 28°C. The light microscopy (80i, Nikon) was used to observe the morphology of the strain after incubation on ISP 2 agar at 28°C for 7–14 days. The Gram staining was performed by standard Gram reaction and confirmed by using KOH lysis (Cerny, 1978). The determination of colony color was done by using the ISCC-NBS color charts (Kelly, 1964). The growth temperature range was tested at 4–40°C at intervals of 4°C on ISP 2 agar. The NaCl tolerance was tested in tryptic soy broth (TSB) and salt concentrations ranging from 0 to 10% (w/v) at intervals of 2%. The pH range for growth was tested in TSB between pH 2.0 and 10.0 at intervals of 1 pH unit. The responses to temperature, pH and NaCl were observed for 14 days. The production of melanoid pigments and catalase activity were determined following protocols described by Lee et al. (2014b). The production of melanoid pigments was examined using ISP 7 medium. Hemolytic activity was assessed on blood agar medium containing 5% (w/v) peptone, 3% (w/v) yeast extract, 5% (w/v) NaCl, and 5% (v/v) horse blood (Carrillo et al., 1996). The plates were examined for hemolysis after incubation at 28°C for 7–14 days. Amylolytic, cellulase, chitinase, lipase, protease, and xylanase activities were determined by growing cells on ISP 2 agar and following protocols as described by Meena et al. (2013). The presence of clear zones around the colonies was taken to indicate the potential of isolates for surfactant production. Antibiotic susceptibility tests were performed by the disc diffusion method as described by Shieh et al. (2003). Antimicrobials used and their concentrations per disc (Oxoid, Basingstoke, UK) were as follows: ampicillin (10 μg), ampicillin sulbactam (30 μg), cefotaxime (30 μg), cefuroxime (30 μg), cephalosporin (30 μg), chloramphenicol (30 μg), ciprofloxacin (10 μg), erythromycin (15 μg), gentamicin (20 μg), nalidixic acid (30 μg), Penicillin G (10 μg), streptomycin (10 μg), tetracycline (30 μg), and vancomycin (30 μg).

### Extract preparation of MUM256

MUM256 was grown in TSB for 14 days prior to fermentation process. The fermentation medium used was FM3 (Hong et al., 2009; Lee et al., 2012). The medium was autoclaved at 121°C for 15 min prior to experiment. Fermentation was carried out in test tubes (30 × 200 mm) containing 20 mL of FM3, at an angle of 45° for 7–10 days at 28°C. The resulting FM3 medium was recovered by centrifugation at 12,000 g for 15 min. The supernatant was filtered and subjected to freeze dry process. Upon freeze-drying, the sample was extracted with methanol for 72 h and the methanol-containing extract was filtered and collected. The residue was re-extracted under the same condition twice at 24 h interval. Subsequently, the methanol-containing extract was evaporated using rotary vacuum evaporator at 40°C. The extract of MUM256 was collected and suspended in dimethyl sulphoxide (DMSO) as vehicle reagent prior to assay.

### Antioxidant activity

#### Free radical scavenging activity determination

DPPH (2,2-diphenyl-1-picrylhydrazyl) radical scavenging assay was performed to determine the antioxidant activity by measuring the hydrogen donating or radical scavenging ability. The DPPH radical scavenging activity of extract of MUM256 was measured according to the previously described method with minor modifications (Ser et al., 2015a). A volume of 5 μL of sample at different concentrations was mixed with 195 μL of freshly prepared 0.016% DPPH in 95% ethanol. The mixture was kept at room temperature in the dark for 20 min before measuring the reduction of DPPH radical at 515 nm with microplate reader. Gallic acid was used as a positive control. The percentage inhibition of DPPH radical or scavenging activity was calculated according to the formula expressed below:

$$\begin{array}{l} \%\text{ DPPH scavenging activity}=\\ \;\frac{\text{Absorbance of control }-\text{ Absorbance of sample}}{\text{Absorbance of control}}\times 100\% \end{array}$$

#### Superoxide anion scavenging activity determination

Superoxide anion scavenging activity/superoxide dismutase (SOD) activity was determined using a commercially available colorimetric microtiter plate method (19160 SOD Assay Kit-WST, Sigma Aldrich) according to the manufacturer's protocol. The SOD activity of MUM256 extract was assayed colorimetrically at 450 nm as the reduction of the Dojindo's highly water-soluble tetrazolium salt, WST-1 (2-(4-iodophenyl)-3-(4-nitrophenyl)-5-(2,4-disulfophenyl)-2H-tetrazolium, monosodium salt) by superoxide anion, ${\text{O}}_{2}^{-}$. Twenty microliter of MUM256 extract at different concentrations were loaded into respective well of the 96-wellplate. The plate was incubated at 37°C for 20 min after the addition of respective reaction solution as the described protocol and prior to measurement of absorbance at 450 nm using a microplate reader. The SOD activity (percentage of inhibition of WST-1 reduction) was determined according to the formula expressed below:

$$\begin{array}{l} \%\text{ SOD activity}=\\ \text{ ​​​​​​}\frac{\begin{array}{cc} \left(\text{Abs control blank }-\text{ Abs buffer blank}\right) & \\ \;-(\text{Abs sample }-\text{ Abs sample blank}) & \end{array}}{\text{Abs control blank }-\text{ Abs buffer blank}}\times 100\% \end{array}$$

Abs = absorbance measured at 450 nm

### Anti-cancer activity

#### Cell lines maintenance and growth condition

All the human cancer and normal cell lines involved in this study was maintained in RPMI (Roswell Park Memorial Institute)-1640 (Gibco) supplemented with 10% fetal bovine serum and 1x antibiotic-antimycotic (Gibco) at 37°C humidified incubator containing 5% CO_2_and 95% air. The cancer cell lines involved were HCT116, HT29, SW480, Caco-2, A549, DU145, CaSki, and MCF-7 while BEAS-2B was used as the normal cell lines in this study (Wong et al., 2012; Goh et al., 2014). The cultures were viewed using an inverted microscope to assess the degree of confluency and to confirm the absence of bacterial and fungal contamination.

#### Anticancer activity determination using MTT assay

The effect of *Streptomyces* sp. MUM256 on cell viability of human cancer cell lines was determined using 3-(4,5-dimethylthiazol-2-yl)-2,5-diphenyltetrazolium bromide (MTT) assay according to the established method with minor modifications (Supriady et al., 2015). Cells were seeded into a sterile flat bottom 96-well plate at a density of 5 × 10^3^ cells/well and allowed to adhere overnight. Twenty microliter of the MUM256 extract was added into each well with the final concentration ranging from 25 to 400 μg/mL. The concentration of DMSO used as the solvent was maintained at 0.05% (v/v) and also incorporated as negative control in all the experiments. Cells were further incubated with the extract for 72 h before performing MTT assay. Twenty microliter of 5 mg/mL of MTT (Sigma) was then added to each well and the plates were incubated at 37°C in a humid atmosphere with 5% CO_2_, 95% air for 4 h. The medium was then gently aspirated, and 100 μL of (DMSO) was added to dissolve the formazan crystals. The absorbance of dissolved formazan product was determined spectrophotometrically at 570 nm (with 650 nm as reference wavelength) using a microplate reader. The percentage of cell viability was calculated as follows:

$$\begin{array}{l} \text{Percentage of cell viability}=\\ \;\frac{\text{Absorbance of treated cells}}{\text{Absorbance of untreated cells }(0.05\%\text{ DMSO only})}\times 100\% \end{array}$$

### Gas chromatography-mass spectrometry (GC-MS) analysis

GC-MS analysis was performed in accordance with our previous developed method with minor modification (Supriady et al., 2015). The machine used was Agilent Technologies 6980N (GC) equipped with 5979 Mass Selective Detector (MS), HP-5MS (5% phenyl methyl siloxane) capillary column of dimensions 30.0 m × 250 μm × 0.25 μm and used helium as carrier gas at 1 mL/ min. The column temperature was programmed initially at 40°C for 10 min, followed by an increase of 3C/min to 250°C and was kept isothermally for 5 min. The column temperature was programmed initially at 40°C for 10 min, followed by an increase of 3°C/min to 250°C and was kept isothermally for 5 min. The MS was operating at 70 eV. The constituents were identified by comparison of their mass spectral data with those standard compounds from NIST 05 Spectral Library (Figure S2).

### Statistical analysis

All the experiments on the antioxidant and cytotoxic properties were performed in quadruplicates. The results were expressed as mean ± standard deviation (SD) and analyzed using SPSS statistical analysis software. One-way analysis of variance (ANOVA) and Tukey's *post-hoc* analysis were performed to determine the significance of difference between the treated and control groups. An independent *t*-test analysis was also conducted to compare between the effect of the extract against cancer and normal cell line. A difference was considered statistically significant when *p* ≤ 0.05.

## Results and discussion

### Phenotypic analyses of strain *Streptomyces* sp. MUM256

Strain MUM256 was Gram-positive and aerobic. The strain grew well on ISP 2, ISP 3, ISP 5, ISP 6, ISP 7 agar, AIA, nutrient agar, and starch casein agar after 1 to 2 weeks at 28°C, whereas it grew poorly on ISP 4 agar. The morphological observation of the 15-day-old culture grown on ISP2 medium revealed an abundance growth of both aerial and vegetative hyphae which was well developed and not fragmented. These morphological characteristics were consistent with its assignment to the genus *Streptomyces* (Williams et al., 1989). The colors of the aerial and substrate mycelium were light yellow and pale yellow on ISP 2 agar. Growth occurred at pH 6.0–10.0 (optimum pH 7.0), with 0–6% NaCl tolerance (optimum 4%) and at 20–40°C (optimum 32°C). Cells were positive for catalase and hemolytic activitiy but negative for melanoid pigment production. Hydrolysis of soluble starch was positive; but negative for hydrolysis of carboxymethylcellulose, tributyrin (lipase), casein, chitin, and xylan. Cells are sensitive to cefuroxime, cephalosporin, chloramphenicol, ciprofloxacin, erythromycin, gentamicin, streptomycin, tetracycline, and vancomycin. Cells are resistant to ampicilin, ampicillin sulbactam, cefotaxime, nalidixic acid, and Penicillin G.

### Phylogenetic and genomic analyses

The almost-complete 16S rRNA gene sequences were determined for strain MUM256 (1343 bp). The 16S rRNA gene sequences of strain MUM256 was aligned with the corresponding partial 16S rRNA gene sequences of the type strains of representative members of the genus *Streptomyces* retrieved from GenBank/EMBL/DDBJ databases. Phylogenetic tree was constructed based on the 16S rRNA gene sequences showed that strain MUM256 (Figure 1) formed a distinct clade with type strains *Streptomyces albidoflavus* DSM 40455^T^, *Streptomyces hydrogenans* NBRC 13475^T^, *Streptomyces somaliensis* NBRC 12916^T^, *Streptomyces koyangensis* VK-A60^T^, and *Streptomyces daghestanicus* NRRL B-5418^T^ at bootstrap value of 72%, indicating the high confidence level of the association (Figure 1). Strain MUM256 exhibited highest 16S rRNA gene sequence similarity to *Streptomyces albidoflavus* DSM 40455^T^ (99.7%), *Streptomyces hydrogenans* NBRC 13475^T^ (99.7%), *Streptomyces somaliensis* NBRC 12916^T^ (99.7%), followed by *Streptomyces koyangensis* VK-A60^T^ (99.5%) and *Streptomyces daghestanicus* NRRL B-5418^T^ (99.5%).

### Antioxidant activity

During metabolism process, organism produces reactive oxygen species as by-products (Cruz De Carvalho, 2008). The accumulation of excess free radicals can result in oxidative stress. It has been associated with many detrimental effects including food deterioration, aging in organisms and cancer promotion (Ames et al., 1993). With the knowledge about the critical role of free oxygen radicals as the etiology of various multifactor diseases such as cancer, neurodegenerative and cardiovascular diseases has prompted investigations on novel and potent antioxidant discovery. Literatures show that plants rich in antioxidants have been extensively studied and reviewed for their protection effects against oxidative stress related disease such as cancer (Wang et al., 2012).

Extensive studies revealed that many potent antioxidative chemical constituents can be derived from microbial origin. The investigation also has evidenced that the microbes derived from extreme environment possess high antioxidant capacity. It was believed that these microbes may have acquired the ability to synthesize specific antioxidative agent or develop specific defense mechanisms after long-term evolutionary processes for survival against oxidative stress (Hong et al., 2009). Likewise, the antioxidant activity of MUM256 extract was investigated by assessing its radical scavenging abilities on both DPPH radicals and superoxide anions. The results are presented in Table 1.

**Table 1:** **The antioxidant activities demonstrated by MUM256 extract in both DPPH assay and SOD activity assay**.

**Concentration of extract *Streptomyces* sp. MUM256 (μg/mL)**	**Antioxidant activities**	
	**DPPH radical scavenging activity (%)**	**Superoxide dismutase activity (%)**
125	ND	16.33 ± 2.89
250	ND	21.35 ± 2.41
500	ND	31.56 ± 4.25
1000	ND	46.45 ± 5.72
2000	6.69 ± 0.83	67.25 ± 8.82
4000	12.08 ± 1.05	NT

*ND, not detected; NT, not tested*.

There are many reports available on the use of DPPH assay in determining the antioxidant activity of *Streptomyces* sp. (Karthik et al., 2013; Lee et al., 2014a), showing that DPPH is widely accepted and well established method for antioxidant activity assessment. DPPH is a discoloration assay using a stable free DPPH radical to assess the free radical scavenging ability of the hydrogen donating antioxidant, which can transfer hydrogen atoms or electron to DPPH radicals. In this study, the color change observed from the purple DPPH radical solution into yellow-colored diphenylpicrylhydrazine suggested that MUM256 extract exhibited hydrogen donating ability at high concentration. The extract MUM265 demonstrated significant (*P* < 0.05) 6.69 ± 0.83% and 12.08 ± 1.05% inhibitions of DPPH activity at both 2 and 4 mg/mL respectively. This result also indicated that the presence of potential antioxidative compounds in the MUM256 extract that can terminate the chain reaction of free radicals.

Furthermore, the dose-dependent manner of superoxide dismutase like activity demonstrated in SOD activity assay further confirming the antioxidant potential of MUM256 extract. It is also important to investigate the ability of the extract to scavenge *in vitro* oxygen-derived species such as superoxide anion (${\text{O}}_{2}^{-}$) because ${\text{O}}_{2}^{-}$ is a powerful oxidants capable to generate more notorious reactive oxygen species, including singlet oxygen, peroxynitrite and hydroxyl radicals (Stadtman and Berlett, 1997) which can result in more serious disease induced by oxidative stress. In this study, the superoxide radical was produced from the hypoxanthine-xanthine oxidase reaction coupled with WST. The MUM256 extract exhibited a potent superoxide anion scavenging activity with significantly strong inhibitory activity (*P* < 0.05) on the formation of yellow water-soluble WST formazan upon reduction with superoxide anion, measured IC_50_ at 1.26 ± 0.17 mg/mL. Strong correlation was reported in previous study between the SOD activity and total phenolic content (Reddy et al., 2012), suggesting that the presence of phenolic compounds in the MUM256 extract.

### Anti-cancer of MUM256 extract

In order to examine the growth inhibitory activity of the MUM256 extract in several human cancer cell lines, MTT assay was employed in this study to measure the cell viability after being treated with the extracts at different concentrations. Furthermore, it has been widely known that genetic background of cell lines could influence the efficacy and sensitivity of anticancer agent. Thus, four human colon cancer cell lines with different molecular characteristics (HCT116, HT-29, Caco-2, and SW480), one human breast cancer cell line (MCF7), one androgen-independent prostatic cancer cell (DU145), one human lung cancer cell line (A549), a human cervical cancer cell line (CaSki) were used as the panel for the anticancer activity screening of the extract. Besides that, the human bronchial epithelium cell line (BEAS-2B) was used to determine the toxicity of the extract against non-cancerous cells in which could reflect the specificity and selectivity of the extract against cancer cells.

**Figure 2:**
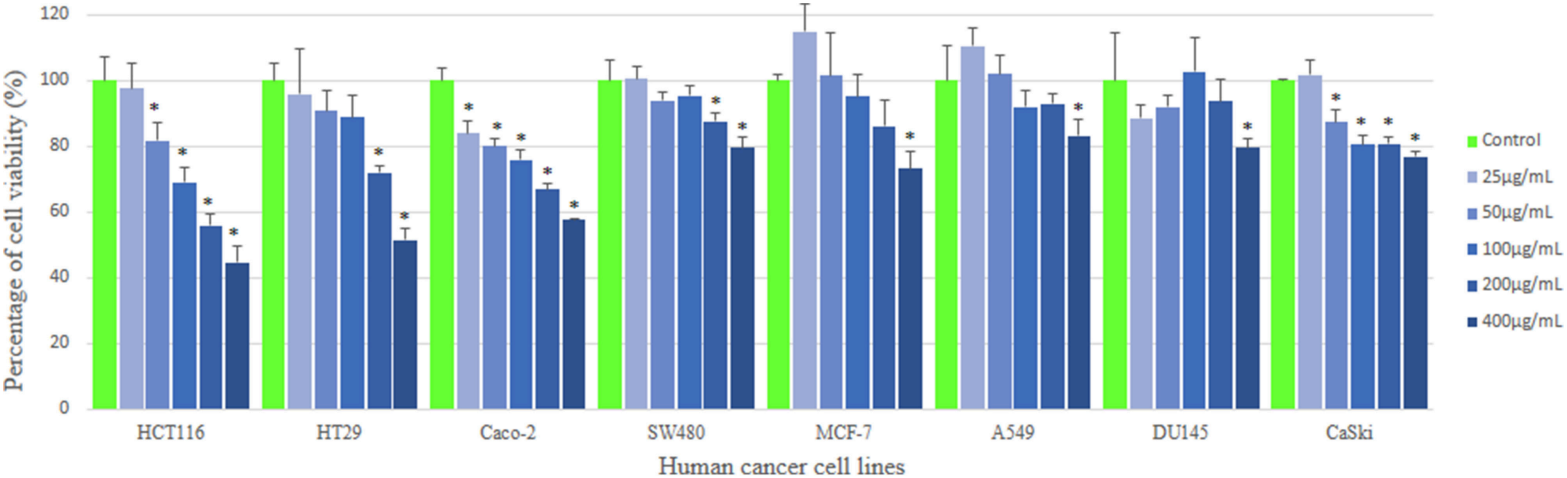
**Anticancer activity of MUM256 extract against human cancer cell lines**. The anticancer activity of streptomyces sp. Mum256 extract against all the cancer cell lines measured using mtt assay. Each bar represents the mean of the cell viability of the cell lines after treatment with extract at respective concentrations tested (*n* = 5). The vertical lines associated with the bars represent the standard deviation of the mean. Symbol (^*^) indicates *p* < 0.05 significant difference compared to control.

**Figure 3:**
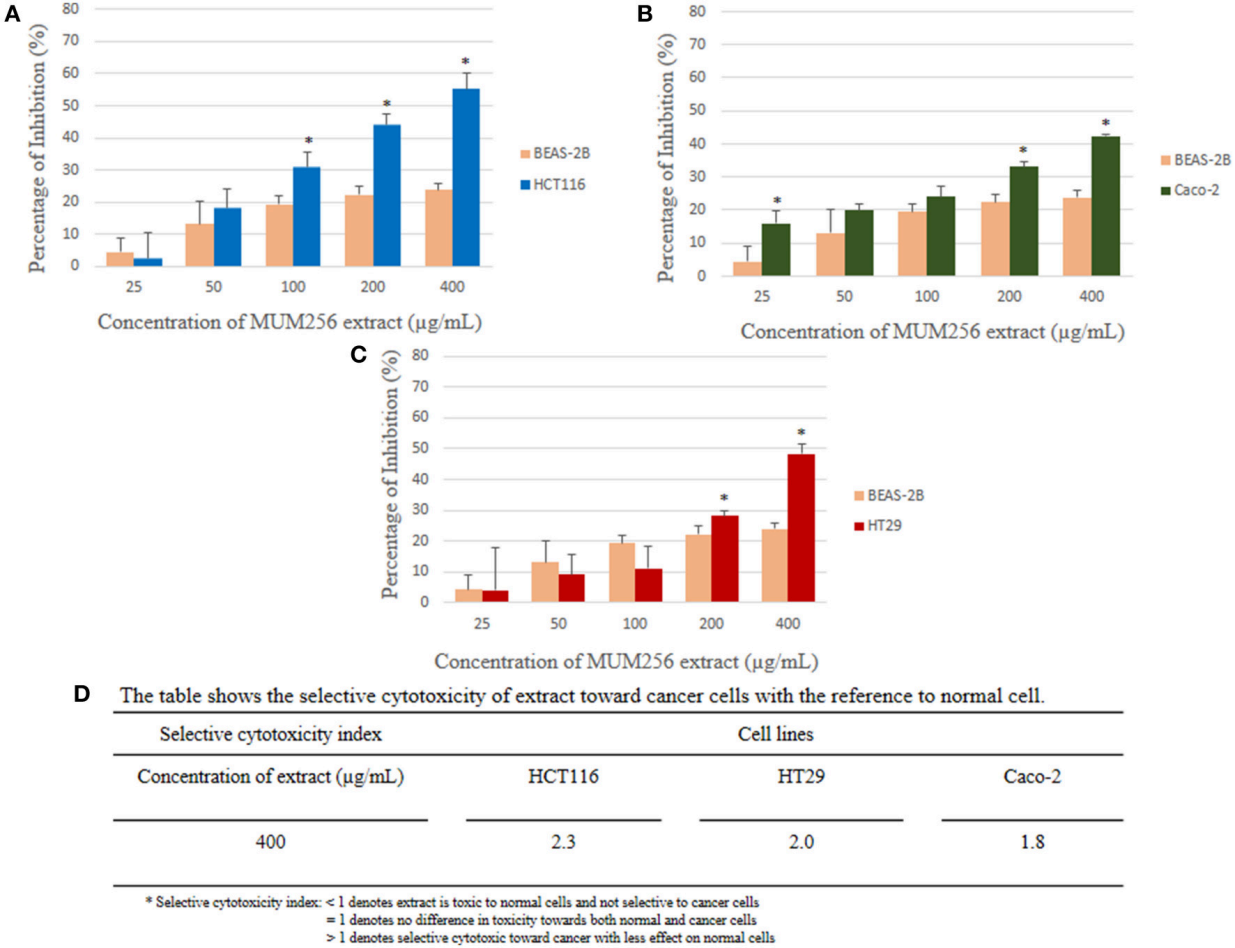
**Comparison of the percentage of inhibition exerted by the extract at respective concentrations between normal cell line (BEAS-2B) and colon cancer cell lines [HCT116 (A), HT29 (B), and Caco-2 (C)]**. Selective cytotoxicity index determined for the extract against the colon cancer cells **(D)**. Each bar represents the mean of inhibition (%) of the extract at respective concentrations tested (*n* = 5) against respective cell lines. The vertical lines associated with the bars represent the standard deviation of the mean. Symbol (^*^) indicates *p* < 0.05 significant difference between the normal cell line and the colon cancer cell line.

MTT assay is used to measure the mitochondrial activity in viable cells based on the activity of mitochondrial dehydrogenase enzyme that reduces the yellow tetrazolium MTT into purple formazan crystal. The amount of the purple formazan formed indicates the number of metabolically active viable cells (Twentyman and Luscombe, 1987). The results of the inhibitory effect of MUM256 extract were illustrated in (Figure 2), showing the cell viability of each cell line after 72 h treatment with different concentration of the extracts. Furthermore, the results were also expressed in term of the selective toxicity of the extract toward HCT116, HT29, and Caco-2 cancer cell lines with the reference to the normal cell BEAS-2B (Figure 3).

Collectively, the MUM256 extract exhibited significant growth inhibitory activity (*P* < 0.05) against all the cell lines tested at the highest concentration (400 μg/mL) when compared to the control. It can be observed that the MUM256 extract exhibited varying levels of inhibitory effect against HCT116, HT29. SW480, Caco-2, A549, DU145, CaSki, and MCF-7 cancer cell lines. Despite that, the extract showed minimal toxic effect on BEAS-2B normal lung cell line with 23.87 ± 2.11% inhibition at 400 μg/mL concentration. In fact, the toxic effect reached a plateau at 100 μg/mL with no significant difference (*P* > 0.05) observed when increased dose to 200 and 400 μg/mL (Figure 3). This result also suggested that the MUM256 extract exhibited a preferential or specific cytoxicity against colon cancer cell line in which HCT116, HT29, and Caco-2 were significantly (*P* < 0.05) inhibited by increased concentration of the extract.

Among the tested panel of cancer cells, HCT116 was the most sensitive cell toward the extract treatment with the IC_50_ measured at 292.33 ± 31.98 μg/mL. With the comparison to the toxic level of the extract determined on BEAS-2B, approximately 2.3-fold significantly stronger cytotoxic effect (*P* < 0.05) against HCT116 was observed at 400 μg/mL (Figure 3). It was then followed by 2.0 and 1.8-fold significant stronger cytotoxic effect (*P* < 0.05) against HT29 and Caco-2 respectively with the reference to BEAS-2B at 400 μg/mL. However, SW480 colon cancer cell appeared less sensitive toward this extract with low cytotoxic effect observed. This could be due to the difference in genetic makeup between those colon cancer cells. The previous investigation demonstrated that SW480 which is a mismatch repair (MMR)-wild type cell line was shown to be more resistant to cytotoxic methylating agent than other colon cancer cells with MMR-deficient cell line such as HCT116 (Liu et al., 1999). Another study also revealed that KRAS G12V mutation conferred resistance in SW480 to chemotherapy with both cetuximab and panitumumab (Kumar et al., 2014). Thus, it was speculated that the cytotoxic effect of the extract may be mediated by MMR-deficiency and wild-type KRAS of colon cancer cell lines.

**Figure 4:**
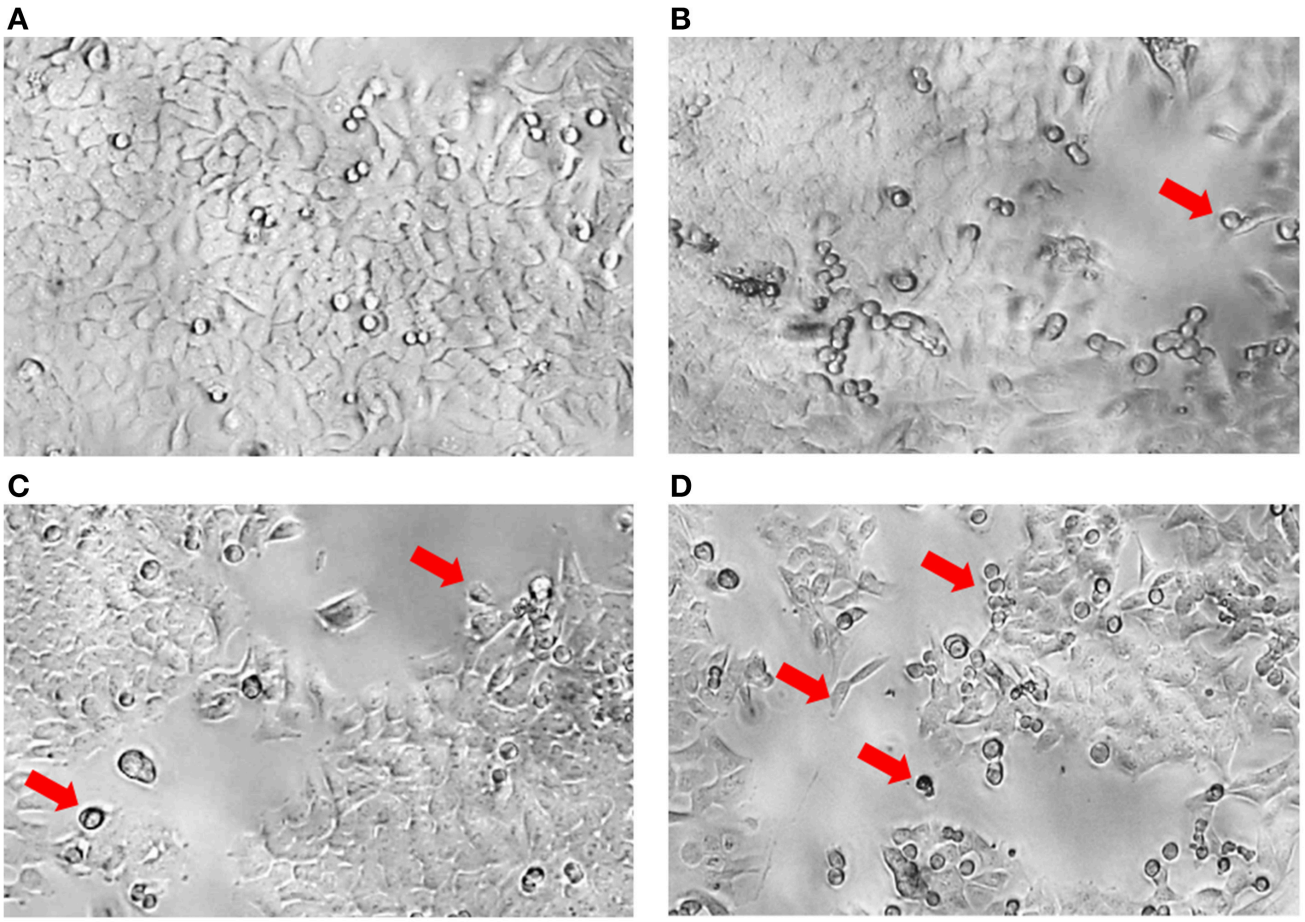
**Morphology of HCT116 after treatment with MUM256 extract at different concentrations**. Comparison of the morphological features of HCT116 after the 72 h with MUM256 extract at respective concentrations [control **(A)**, 100 μg/mL **(B)**, 200 μg/mL **(C)**, 400 μg/mL **(D)**] observed under an inverted microscope with objective lens x40. Arrow indicates the abnormal morphological features resulted from the anticancer effect of MUM256 extract.

Although significant results were demonstrated in this study indicating that the MUM256 extract exhibited certain extent of cytotoxic effect on colon cancer cell line, it should be noted that using MTT assay is not possible to differentiate between cell growth inhibition and an increase in cell death. In Figure 4, most of the HCT116 appeared as normal angular and spindle shapes in control (a), but most of the cells lost these features after treated with increasing concentrations of the extract (b, c, and d). For instance, cell shrinkage with lesser cytoplasm mass and even apoptotic bodies can be observed (indicated by arrows) in Figures 4B–D. These morphological changes of the cells observed after treated with the extract has provided some insight on the effect of the extract against the HCT116. However, data from studies focusing on elucidation of the molecular basis is essential in order to determine the putative anticancer activity of the extract against colon cancer cells.

### GC-MS analysis of MUM256 extract

**Figure 5:**
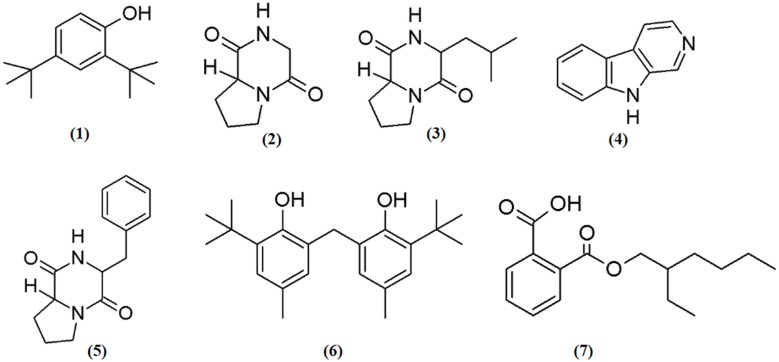
**Chemical structures of constituents detected in MUM256 extract**.

In the present investigation, the MUM256 extract has shown significantly antioxidant capacities in SOD activity and DPPH assays and anticancer properties against human colon cell lines. In this regards, it has prompted the necessities to perform chemical constituents profiling of the MUM256 extract. Hence, GC/MS analysis was employed to identify the chemical constituents present in the extract. The analysis revealed that the presence of phenolic, pyrrolopyrazine, β-carboline and dicarboxylic acid ester compounds in the MUM256 extract. The detailed information about the identified chemical constituents were listed in Table 2 and the chemical structures were illustrated in Figure 5. Furthermore, the mass spectrum of the constituents identified by GC/MS in MUM256 extract is also provided in Figure S2.

**Table 2:** **Chemical constituents identified in of ***Streptomyces*** sp. MUM256 extract**.

**No**.	**Constituents**	**Class**	**Retention time (min)**	**Molecular formula**	**Molecular Weight**	**Similarity (%)**	**References**
1	Phenol,2,4-bis(1,1-dimethylethyl)-	Phenolic compound	44.445	C_14_H_22_O	206	96	Narendhran et al., 2014
2	Pyrrolo[1,2a]pyrazine-1,4-dione,hexahydro-	Pyrrolopyrazine	53.297	C_7_H_10_N_2_O_2_	154	98	Narasaiah et al., 2014; Ser et al., 2015a
3	Pyrrolo[1,2a]pyrazine-1,4-dione,hexahydro-3-(2-methylpropyl)-	Pyrrolopyrazine	58.510	C_11_H_18_N_2_O_2_	210	64	Narasaiah et al., 2014; Abdullah et al., 2015; Manimaran et al., 2015; Ser et al., 2015a
4	9*H*-Pyrido[3,4-b]indole	β-carboline alkaloid	60.381	C_11_H_8_N_2_	168	96	Zheng et al., 2006
5	Pyrrolo[1,2-a]pyrazine-1,4-dione,hexahydro-3-(phenylmethyl)-	Pyrrolopyrazine	72.071	C_14_H_16_N_2_O_2_	244	97	Narasaiah et al., 2014
6	Phenol,2,2′-methylenebis[6-(1,1-dimethylethyl)-4-methyl-	Phenolic compound	73.507	C_23_H_32_O_2_	340	96	Narendhran et al., 2014
7	1,2-Benzenedicarboxylic acid, mono(2-ethylhexyl) ester	Dicarboxylic acid ester	76.883	C_16_H_22_O_4_	278	91	Krishnan et al., 2014

Phenolic compounds have been widely known as potent antioxidant agents or free radical terminators which they possess hydrogen-donating ability to reduce free radicals (Sulaiman et al., 2011; Yogeswari et al., 2012). Phenol,2,4-bis(1,1-dimethylethyl)- (1), phenol,2,2′-methylenebis[6-(1,1-dimethylethyl)-4-methyl- (6) were the two phenolic compounds identified from the extract. Similarly, a recent study showed the detection phenol,2,4-bis(1,1-dimethylethyl)- (1) with GC/MS in *Streptomyces cavouresis* KUV39 isolated from vermicompost samples in India and demonstrated that this compound exhibited potent antioxidant properties and cytotoxicity against Hela cells (Narendhran et al., 2014). Thus, *Streptomyces* sp. MUM256 could be also a potential source of phenolic compounds to be used as preventive agent for oxidative-stress related diseases.

In the present study, the detected three pyrrolopyrazine compounds include, pyrrolo[1,2a]pyrazine-1,4-dione, hexahydro- (2), pyrrolo[1,2a]pyrazine-1,4-dione, hexahydro-3-(2-methylpropyl)- (3), and pyrrolo[1,2-a]pyrazine-1,4-dione,hexahydro-3-(phenylmethyl)- (5) were also present in previously isolated *Streptomyces* sp. (Narasaiah et al., 2014; Manimaran et al., 2015; Ser et al., 2015a). Both of the pyrrolopyrazine compounds identified had been suggested to possess potent antioxidant activity (Ser et al., 2015a). Besides the detection of pyrrolopyrazine in *Streptomyces*, Gopi et al. (2014) also reported that the structure of pyrrolo[1,2a]pyrazine-1,4-dione, hexahydro- (2) isolated from sponge associated *Bacillus* sp. has the ability to reduce oxidative damages by radicals. Furthermore, another study revealed that the extract of *Micrococcus lutues* containing hexahydro- (2) and pyrrolo[1,2a]pyrazine-1,4-dione, hexahydro-3-(2-methylpropyl)- (3) exhibited promising cytotoxic effect on HCT15 with (Abdullah et al., 2015). Thus, it was suggested that both of the identified pyrrolopyrazine could have contributed the antioxidant and anticancer activities observed in MUM256 extract.

Furthermore, a tricyclic indole β-carboline alkaloid, 9*H*-pyrido[3,4-b]indole (4) was detected in MUM256 extract. Previous study by Zheng et al. (2006) demonstrated that this compound which is also known as norharman extracted from a marine bacterium, *Pseudoalteromonas piscicida*, exhibited cytotoxicity toward both HeLa cervical cancer and stomach cancer cells with an IC_50_ of 5 μg/mL. It was shown that norharman caused HeLa cells death via apoptotic process, specifically through the perturbation of cell cycle at G_2_M phase of the cancer cell (Zheng et al., 2006).

Lastly, 1,2-benzene dicarboxylic acid, mono 2-ethylhexyl ester (7) has been detected in various sources ranging from plant extracts (Akpuaka et al., 2012; Sivasubramanian and Brindha, 2013), endophytic fungal (Verma et al., 2014), and also microbial origin including *Streptomyces* sp. (Krishnan et al., 2014). In previous study, the cytotoxicity of 1,2-benzene dicarboxylic acid, mono 2-ethylhexyl ester (7) extracted from *Streptomyces* sp. was evaluated against liver cancer cell line HepG2 and also breast cancer cell line MCF7 with IC_50_ at 42 and 100 μg/mL respectively (Krishnan et al., 2014).

According to the GC/MS analysis, the identified chemical constituents are well recognized for their antioxidant and anticancer activity and we postulate that these constituents could be the major contributing factor for both antioxidant capacity and anticancer activities of MUM256 extract.

## Conclusion

In summary, the findings demonstrates that MUM256 extract exhibits antioxidant and anticancer activities. The extract is able to scavenge superoxide anion radicals in dose dependent manner and show a selective cytotoxic effect toward colon cancer cells. The phenolic compounds, pyrrolopyrazine, β-carboline and dicarboxylic acid ester present in the extract could be responsible for the antioxidant and anticancer activities observed. Those findings suggest that *Streptomyces* sp. MUM256 could be potential source for antioxidative agents and hence merit further studies concerning the development of chemopreventive drugs against cancer.

### Conflict of interest statement

The authors declare that the research was conducted in the absence of any commercial or financial relationships that could be construed as a potential conflict of interest.
