# Supplementary material for: Investigation of Antioxidative and Anticancer Potentials of Streptomyces sp. MUM256 Isolated from Malaysia Mangrove Soil
Source: Front Microbiol. 2015 Nov 26;6:1316. doi: 10.3389/fmicb.2015.01316 (PMC4659911; doi:10.3389/fmicb.2015.01316)
Supplement: Supplementary file 1 [file Image1.PDF]

Figure S1. Maximum-likelihood tree based on almost complete 16S rRNA sequences (1343 nucleotides) showing relationship between strain MUM256 and representatives of some other related taxa. Bootstrap values (>50%) based on 1000 re-sampled datasets are shown at branch nodes. Bar, 0.002 substitutions per site. Asterisks indicate that the corresponding nodes were also recovered using maximum-likelihood tree-making algorithms.

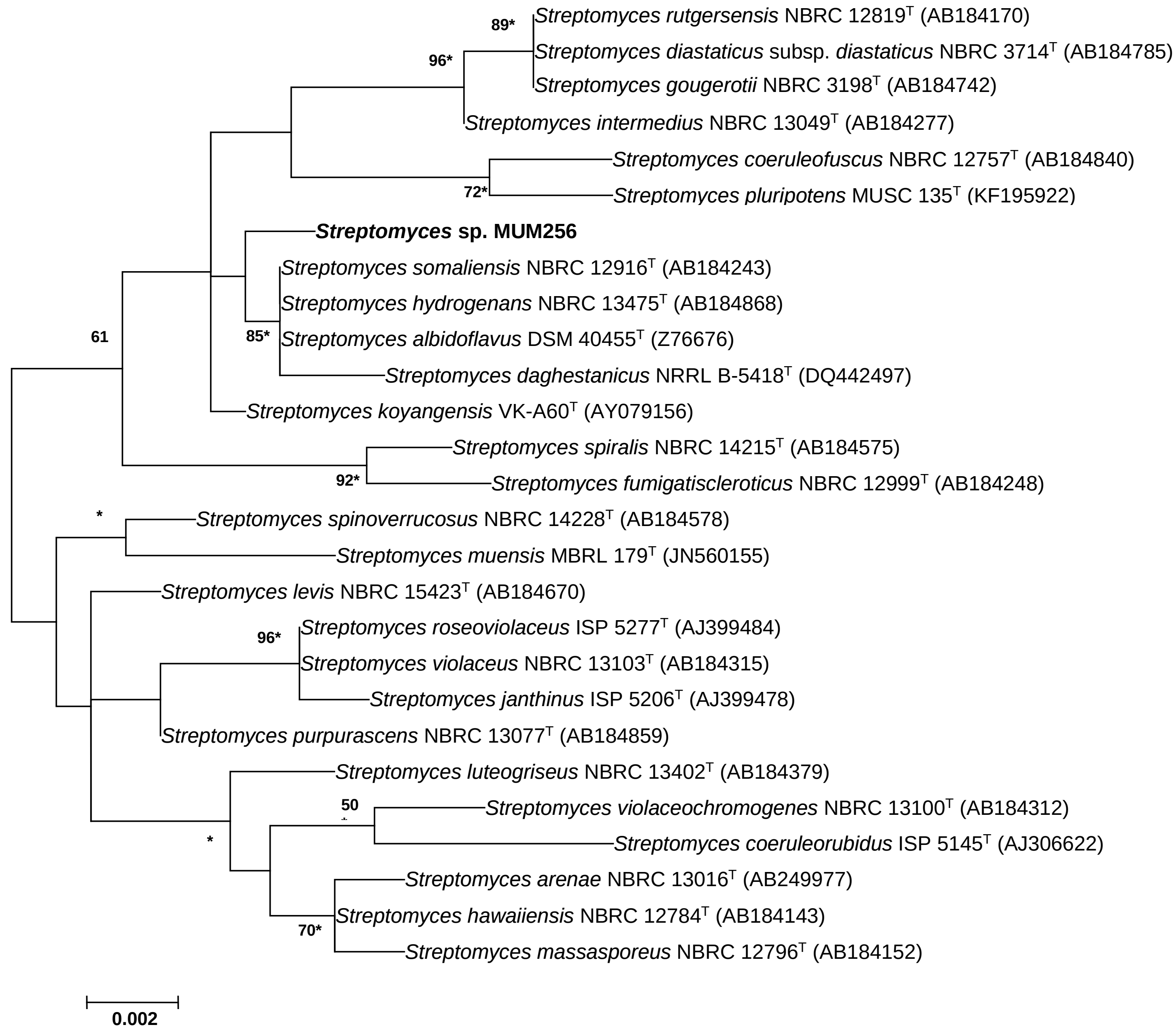

Figure S2. The mass spectrum of the constituents **(1-7)** identified from the GC/MS analysis. (a) The mass spectrum of the constituents obtained from the MUM256, (b) the mass spectrum of the standard compounds available on NIST05 Spectral Library.

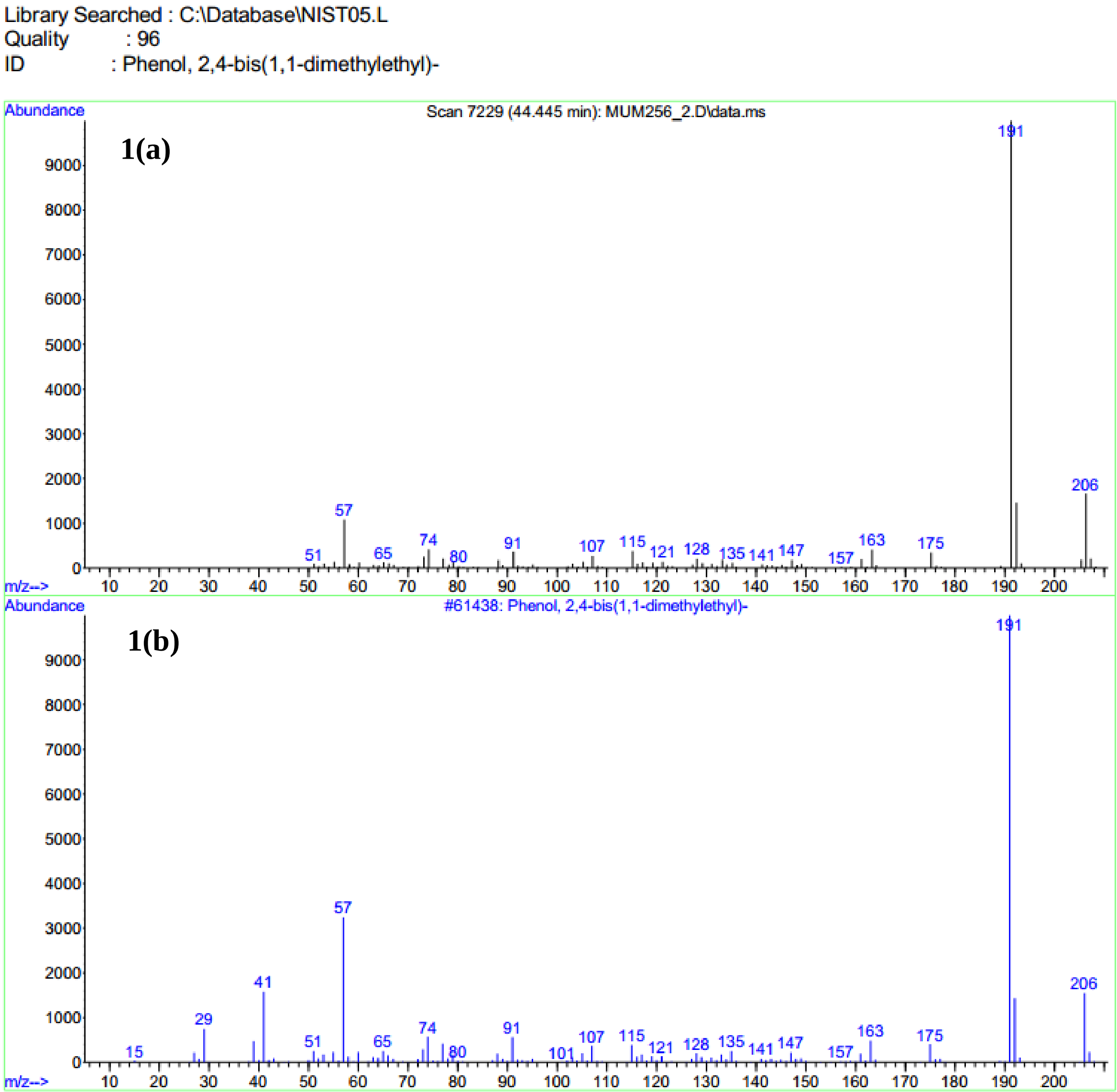

Library Searched : C:\Database\NIST05.L
Quality : 98
ID : Pyrrolo[1,2-a]pyrazine-1,4-dione, hexahydro-

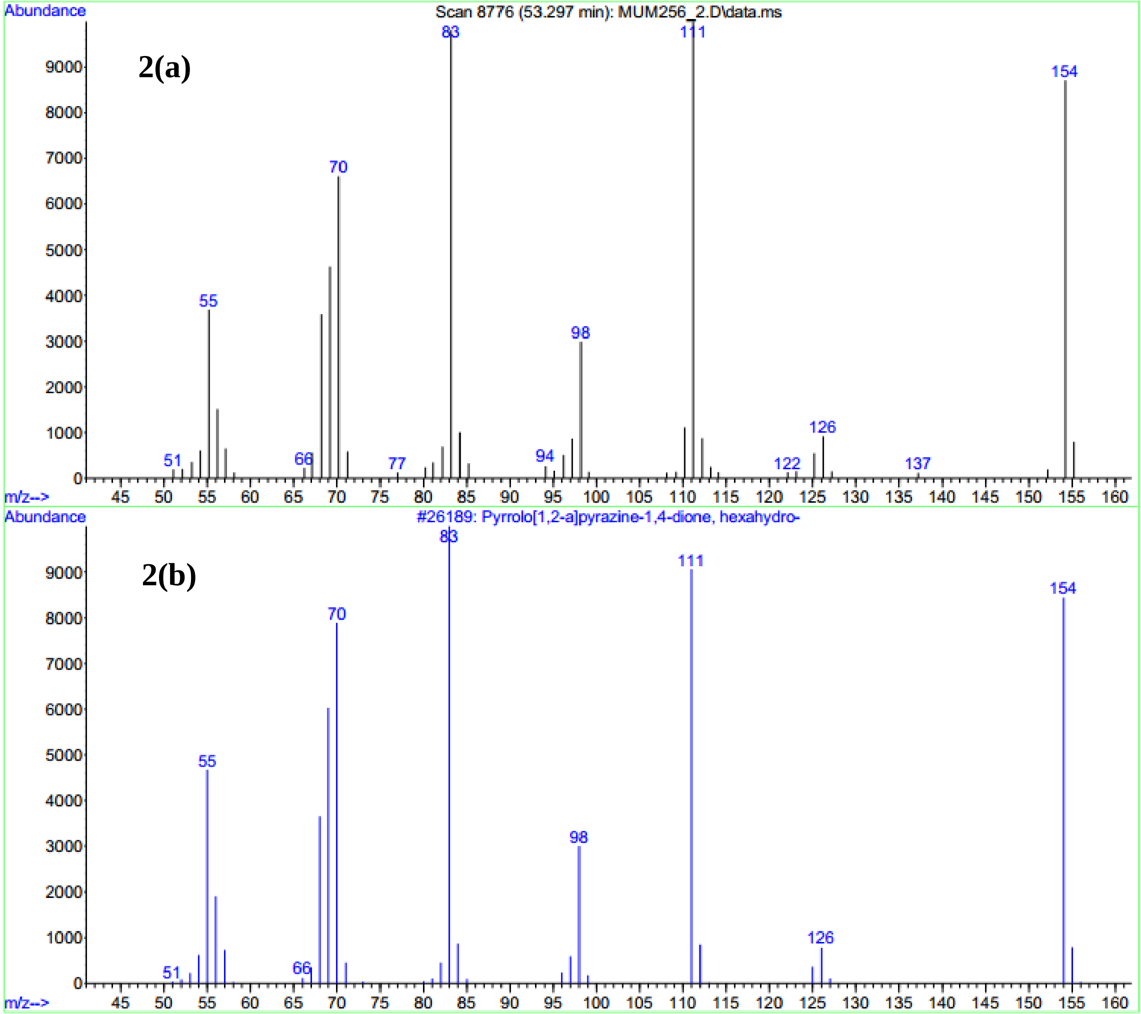

46

Library Searched : C:\Database\NIST05.L
Quality : 64
ID : Pyrrolo[1,2-a]pyrazine-1,4-dione, hexahydro-3-(2-methylpropyl)-

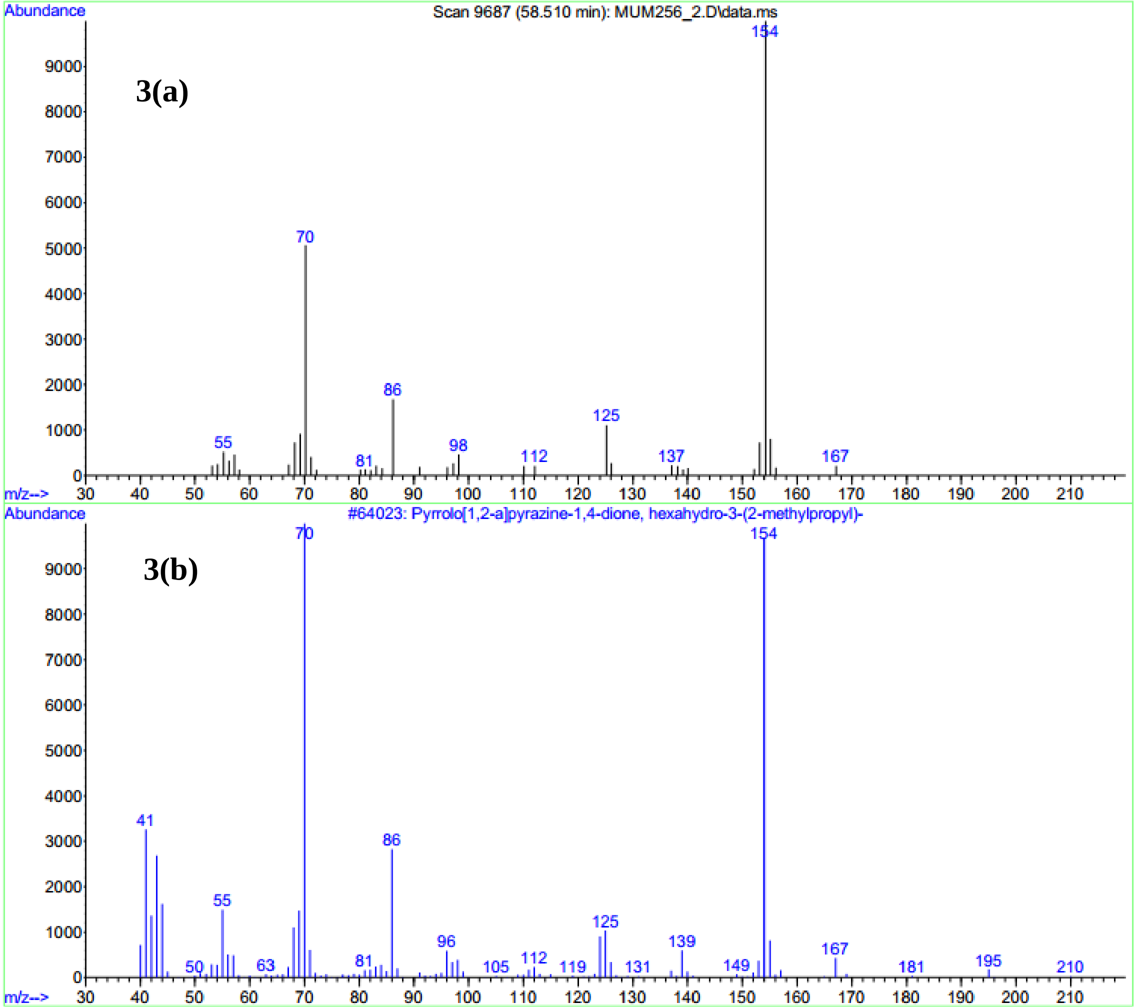

Library Searched : C:\Database\NIST05.L
Quality : 96
ID : 9H-Pyrido[3,4-b]indole

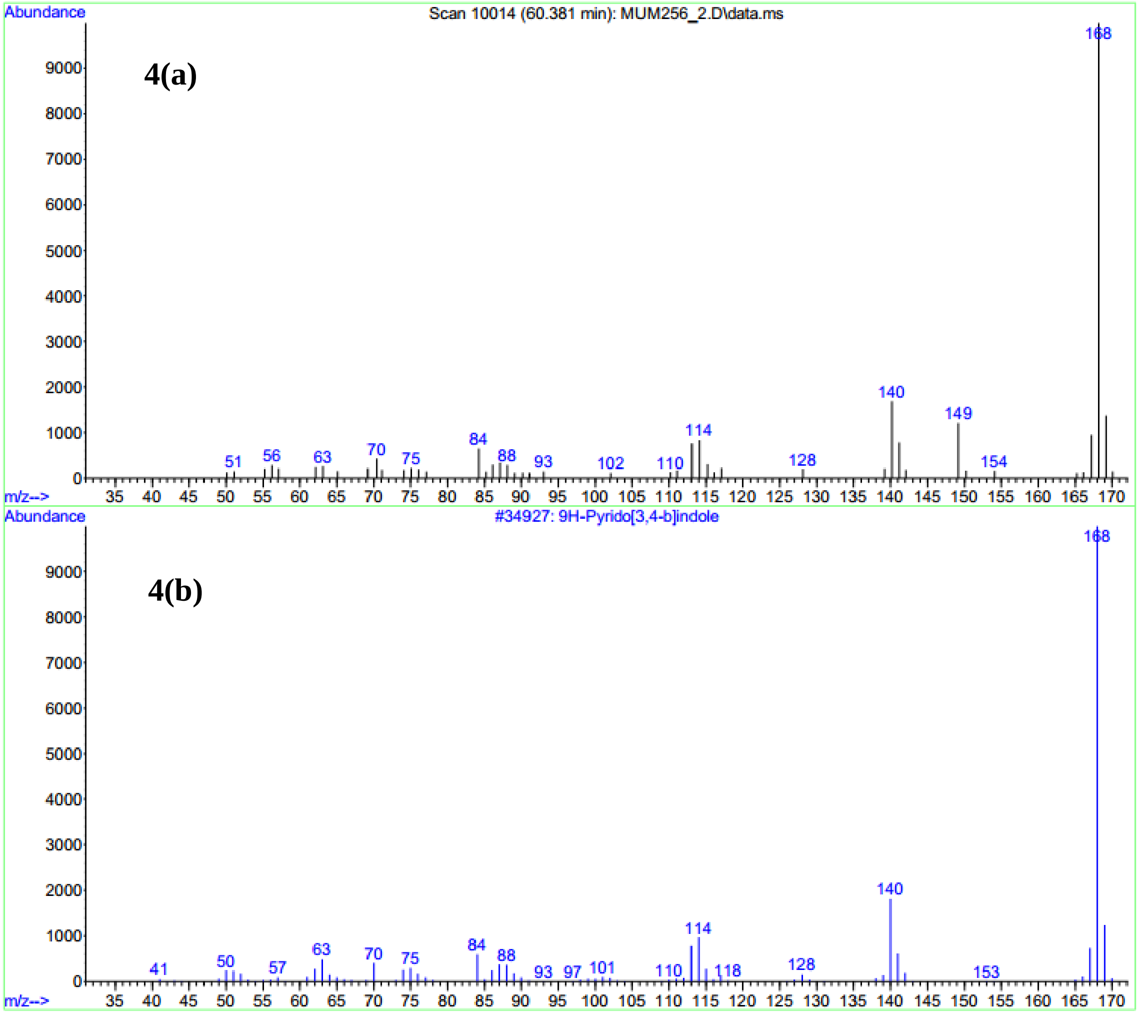

48

Library Searched : C:\Database\NIST05.L
Quality : 97
ID : Pyrrolo[1,2-a]pyrazine-1,4-dione, hexahydro-3-(phenylmethyl)-

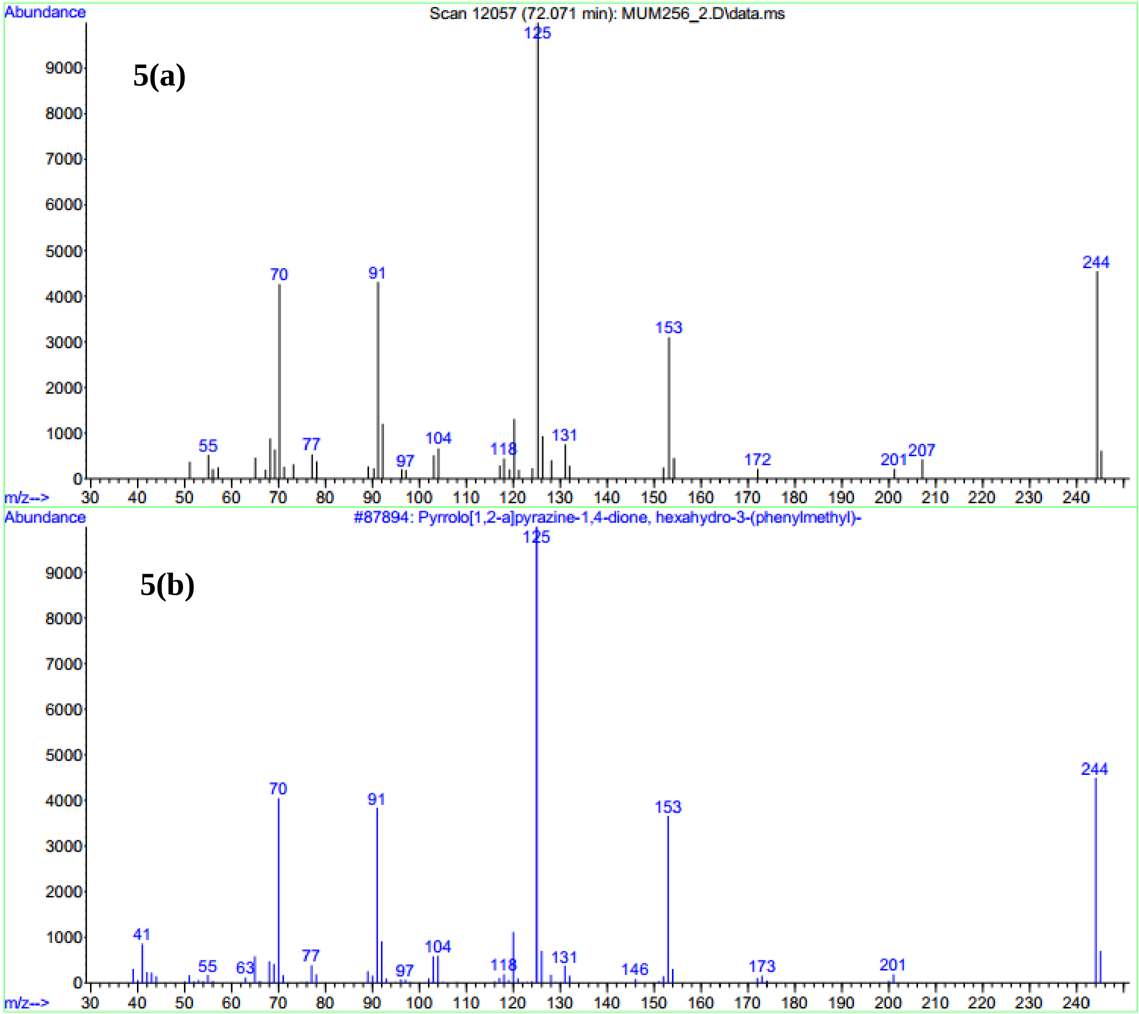

49

Library Searched : C:\Database\NIST05.L
Quality : 96
ID : Phenol, 2,2'-methylenebis[6-(1,1-dimethylethyl)-4-methyl-

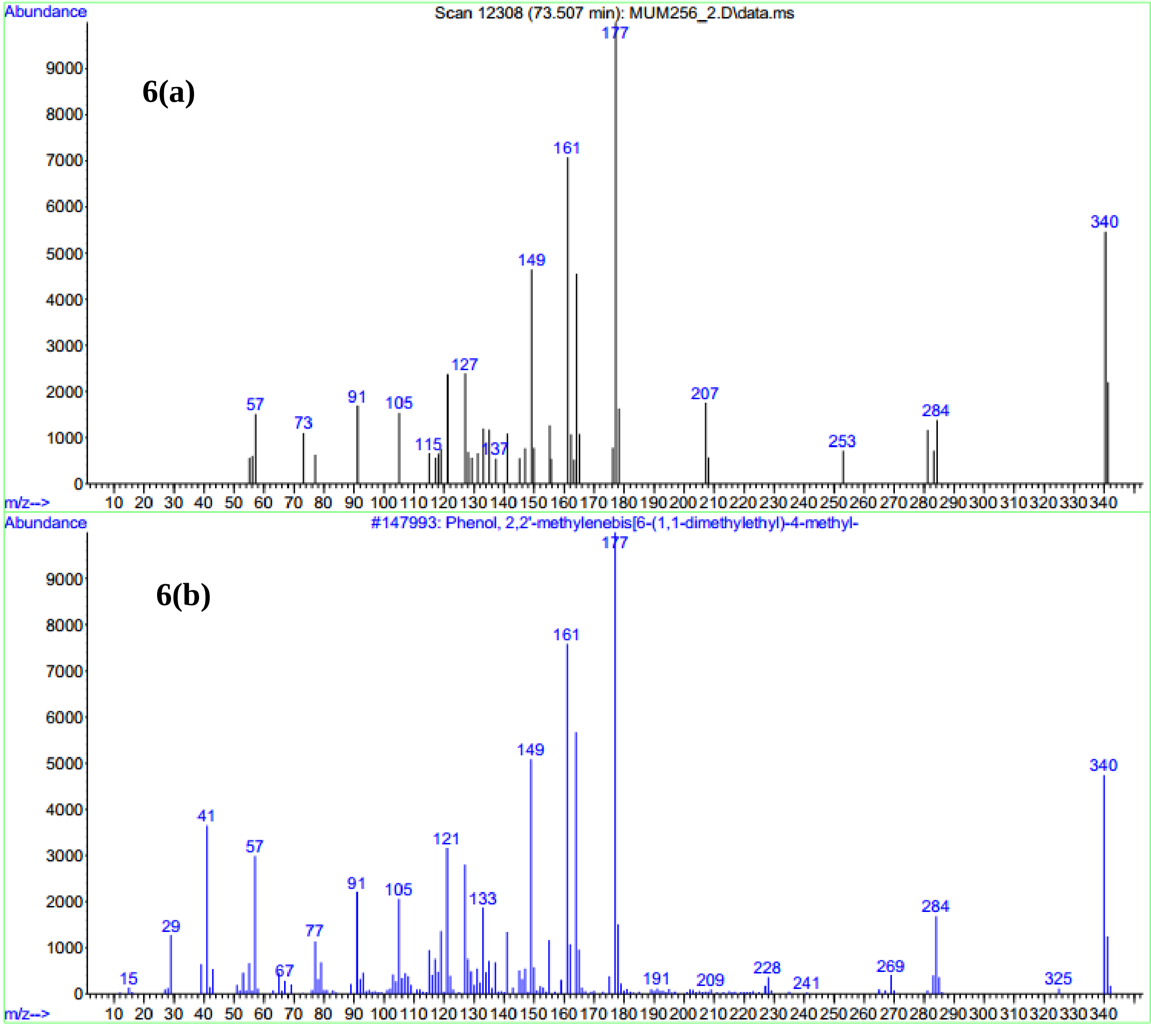

Library Searched : C:\Database\NIST05.L
Quality : 91
ID : 1,2-Benzenedicarboxylic acid, mono(2-ethylhexyl) ester

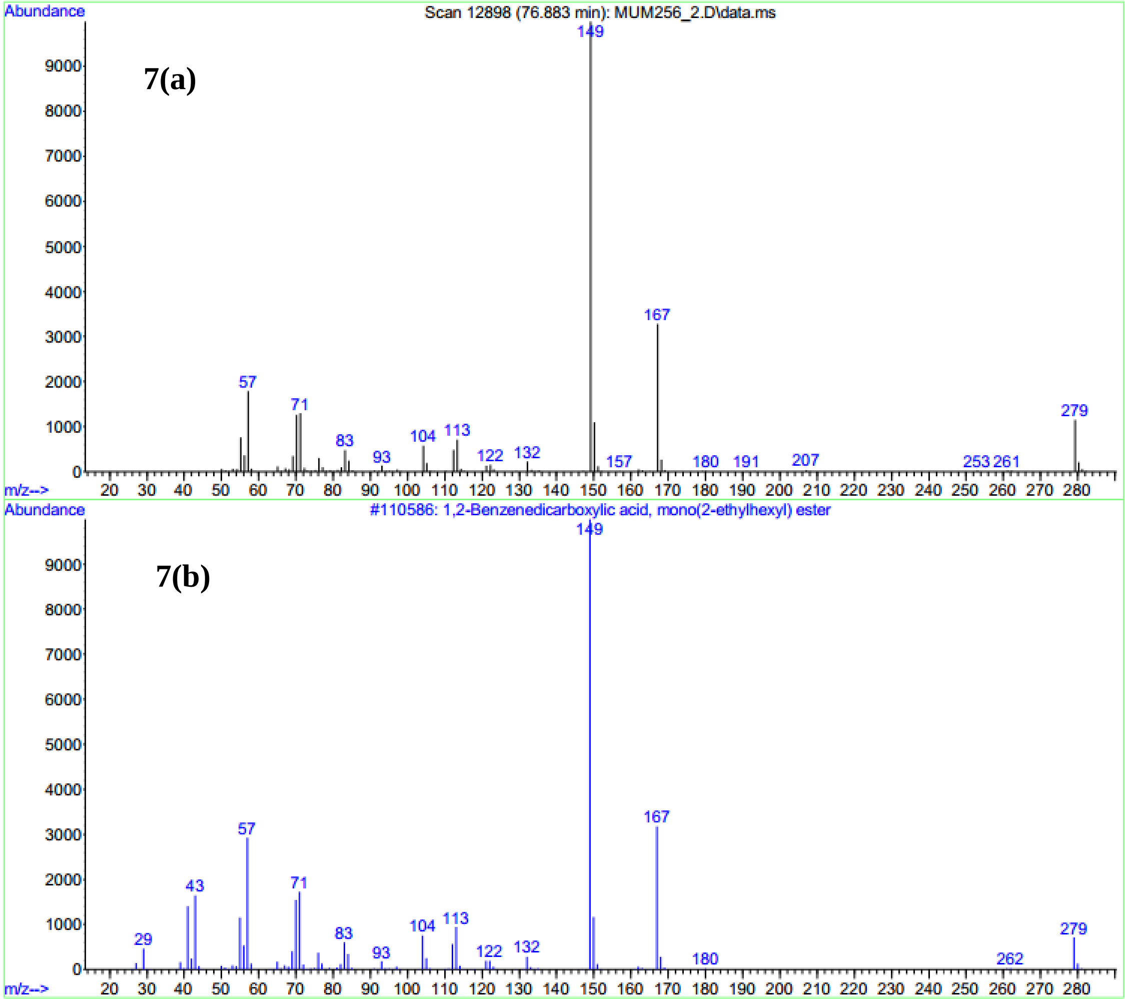

Tan Loh Teng-Hern, Hooi-Leng Ser, Wai-Fong Yin, Kok-Gan Chan, Learn-Han Lee*, Bey-Hing Goh* (2015). Investigation of antioxidative and anticancer potentials of *Streptomyces* sp. MUM256 isolated from Malaysia mangrove soil. *Frontiers in Microbiology*

*Correspondence: Goh Bey-Hing, E-mail: goh.bey.hing@monash.edu and Lee Learn-Han, E-mail: lee.learn.han@monash.edu; leelearnhan@yahoo.com and. Affiliation: Monash University Malaysia.
